# Leveraging artificial intelligence to assess the impact of COVID-19 on the teacher-student relationship in higher education

**DOI:** 10.1371/journal.pone.0317567

**Published:** 2025-03-24

**Authors:** Md Juwel Ahmed Sarker, Mahmudul Hasan, Alamgir Kabir, Abdullah Haque

**Affiliations:** 1 Department of Development Studies, Hajee Mohammad Danesh Science and Technology University, Dinajpur, Bangladesh; 2 Department of Computer Science and Engineering, Hajee Mohammad Danesh Science and Technology University, Dinajpur, Bangladesh; 3 Department of Sociology, Hajee Mohammad Danesh Science and Technology University, Dinajpur, Bangladesh; 4 Department of Mathematics, Hajee Mohammad Danesh Science and Technology University, Dinajpur, Bangladesh; National University of Lesotho, LESOTHO

## Abstract

The teacher-student relationship has far-reaching implications for educational outcomes at the tertiary level. Teachers contribute to students’ success in various ways, including academic support, career counseling, personal mentoring, etc., that help them succeed academically and professionally. COVID-19 disrupted teacher-student interaction and hindered the flow of teacher’s support to students. The damage caused by the pandemic to the higher education sector has mostly recovered. However, the trusting relationship between teacher and student is yet to get back to a pre-pandemic stage. Using stratified sampling technique, we collected nationally representative data from university students in Bangladesh and examined the relationship between COVID-19 and various aspects of the teacher-student relationship. We also explored the association between aspects of the teacher-student relationship and academic outcomes. In our sample, 28% of respondents are from STEM, and 72% are from non-STEM academic disciplines. We employed a subset of Artificial Intelligence (unsupervised machine learning) algorithms K-Modes clustering and Non-negative matrix factorization to cluster the data according to its internal structure. We created a new analysis technique called Absolute Rate of Fluctuation (ARF) to identify the fluctuations between the variables. ARF can track the fluctuations in any relationship induced by undesirable events such as the COVID-19 outbreak. We observed a deterioration in the interaction between teachers and students during COVID-19. However, the class conduction, exam taking, and assessment system were the most affected areas compared to personal interaction, catering support to students, and collaborative research activities.

## Introduction

COVID-19 had a detrimental effect on several areas of the education industry, particularly university teaching [1]. Frequent disruptions occurred with respect to traditional pedagogy, the assessment system, and overall learning environment. It led to a drastic transformation in education technology and other areas of teaching and learning, which has profoundly impacted students’ academic outcomes. The vulnerability of developing countries was disproportionately high and devastatingly affected students’ learning [2]. Many educational institutions lacked the necessary technology and other resources to continue academic activities online [3]. Using samples of 30,383 students from 62 countries on the impact of the pandemic, [4] found that globally, 86.7% of students had to shift to online classes. However, 59.9% of African and 58.2% of Asian students did not have an Internet connection [4]. Thus, educational institutions remained closed in many countries throughout the pandemic period.

COVID-19 altered students’ social and emotional lives worldwide [5]. The prevalence of anxiety and depression increased tremendously during that time [6]. Because of this devastating mental health condition, the number of suicides has also increased in various countries [7]. Due to COVID-19, students and teachers suffered mental health-related issues that impact their relationships more significantly. According to [8] among university teachers in Bangladesh, 35.4% went through depression, 43.7% anxiety, and 6.6% stress, respectively, during the pandemic. [9] observed similar findings for German teachers and principals that they were exhausted and stressed during COVID-19. Furthermore, COVID-19 also affected teachers job-specific passions. [10] conducted a study on the pandemic’s impact on Chinese rural teachers’ job sentiments and saw that teachers’ passion for the teaching profession has reduced due to teaching stress and career-related stress that arose during COVID-19.

The depressing mental health conditions induced by the pandemic also affected other socioeconomic domains of teachers’ and students’ lives. It reduced teachers’ productivity, and willingness to conduct online classes and support students during the pandemic. We also saw that teachers were not optimistic regarding online teaching. [11] conducted a study in Hong Kong that shows that teachers were more pessimistic about online teaching than students. Pessimism regarding online teaching among teachers motivated many not to conduct classes during the pandemic, ultimately leading to sessions jot in many public universities in developing countries like Bangladesh. [12] surveyed 1000 individuals to identify triggering factors of mental stress among university students during COVID-19 and found that 80.6% of private university students and 77% of public university students in Bangladesh are stressed due to tuition-related and session jam-related anxiety, respectively. It is likely that session jams, academic discontinuity, and career uncertainty, which are the core reasons for mental stress for graduate students, may harm the teacher-student relationship at the university level. Though the teacher-student relationship in higher education is under research issues, it has great relevance for the academic outcome [13]. Therefore, it is crucial to explore the impact of COVID-19 on the teacher-student relationship at the university level. To find the relationship and the effects of COVID-19 on it, we investigate the datasets, and our contribution to this paper are:

- To determine the nature of interactions between teachers and students, we use unsupervised machine learning clustering algorithms on primary data. To better convey the linear nature of this relationship, we segment it into four distinct clusters.
- We examine the relationship among the pre-COVID-19, during COVID-19, and post-COVID-19 periods, as well as COVID-19’s impact on the teachers students relationship. Additionally, in the inter-division of the relationship, we examine how the relationship varies based on gender, academic division, and period change.
- To figure out how far an event can cause a change in a relationship, we created a new approach called Absolute Rate of Fluctuation (ARF) as well as we find out the building block variables that are mostly to criticize for the change in the relationship, and we talk about how to reconstruct it.

The following parts of this study are structured as follows: In Sect 2, we look at the previous works in this field. In Sect 3, we examine the methods and materials. Sect 4 contains the analysis and discussion of the results. Last, Sect 5 provides the conclusion and future work.

## Literature review

Many researchers investigated whether the immediacy of the teacher predicts the effectiveness of teaching [14]. They conceptualized immediacy as nonverbal behaviors that reduce physical, and psychological distance between teachers and students and teaching effectiveness as the ability of a teacher to produce an effective behavioral and cognitive environment congenial for student learning. The researchers found that teacher immediacy is associated with a 46% higher likelihood that students would like their course instructor and a 20% greater probability that they would enjoy the course content. Immediacy is also related to an 18% increase in students’ behavioral commitment.

A good teacher-student relationship facilitates the growth of both instructors and students, especially when it helps pupils make socio-emotional, behavioral, and academic adjustments. It is also a predictor of students’ academic performance. [15] sees students as the primary consumers of education, and professors stimulate the development of students’ intellectual faculty by encouraging their opinions and qualities. Effective advisor-student communication influences student happiness and prevents academic challenges, resulting in improved educational assistance. [16] found that fostering a calm and enjoyable learning environment and developing harmonious teacher-student relationships help students overcome emotional barriers and learning anxiety. It also improves their learning motivation and autonomous learning consciousness.

The author delved further into the following questions: 1) What kind of teacher-student interaction do students want? and 2) What do students consider to be good teaching standards? The author found that most students consider effective teachers adept at developing positive relationships with their students, engaging in conversation, and comprehending students’ academic circumstances.

According to [17], teacher-student interaction is associated with learning, classroom management, and student absenteeism. They significantly influence both teachers’ and students’ experiences in higher education. According to [18], analyzing teacher-student relations in the context of higher education is crucial for at least three following reasons:

First, significant human and financial consequences are associated with student dropout rates worldwide. Improving the teacher-student relationship can help reverse this unfavorable tendency. Thus, researching teacher-student relationships is pertinent.

Second, university instructors are impacted by the need to fit in. Therefore, as relational approaches to teaching propose, a healthy “relational classroom environment,” involving pleasant interactions and relationships, may also have favorable impacts on the teachers themselves.

Thirdly, the significance of the teacher-student relationship necessitates thorough consideration given the rising priority accorded to excellence in university teaching as part of the discourse on “Scholarship in Teaching and Learning.” To facilitate excellence in teaching and learning at universities, it is important to investigate the nature, establishment, and consequences of social elements like teacher-student relationships in greater detail.

Teacher-student relationships can be considered a prerequisite for all students’ successful learning. Still, they seem especially important for at-risk students in terms of decisions on whether to continue their studies or drop out. The need to understand and prevent student dropout has significantly driven the development of these teacher-student relationship studies in higher education. Researchers have focused on the extent of academic and social integration as a deciding factor in whether students stay at or leave university, mainly drawing on Tinto’s framework (1975) of student dropout.

Still, the number of studies on the teacher-student relationship in the context of higher education is limited. Researchers less comprehensively and systematically examine this issue [18]. Furthermore, studies conducted on teacher-student relationships often lack sound theoretical and conceptual rigor. Thus, there is a knowledge gap regarding teacher-student relations in higher education. The current research explores various dimensions of teacher-student relationships, which will help reduce the knowledge gap.

COVID-19 has impacted the teaching and learning paradigm significantly. It has affected university teaching and learning in at least two ways. Firstly, it disrupted the regular academic activities of almost every university worldwide, and many could not provide educational facilities for the students. Even so, there was a considerable deficit in the online educational system. Universities in developing countries are the worst victims in this regard. In many developing countries, universities could not smoothly operate regular classes, exams, and other academic activities during the pandemic. Some universities tried to conduct academic activities online but struggled with infrastructural backwardness, while others remained shut throughout the crisis. In particular, most Bangladeshi universities remained closed during the entire COVID-19 period. This academic deadlock led to the academic year loss of the students and, subsequently, strained teacher-student relationships.

Secondly, COVID-19 disturbed the teacher-student support mechanism for university graduates. Teachers typically provide students with various supports, such as career guidance, research supervision, mental health-related support, personal mentoring, and so on, to help them cope with adversity. However, COVID-19 hampered those supportive activities and disrupted oﬄine communication between students and teachers.

Furthermore, Atle Fretheim et al. (2021) performed a cohort study among students in higher education in Norway [19]. An uncorrected bi-variate analysis was performed using a sample of 26754 students across 14 different institutes of higher learning to determine whether in-person or online instruction was used. The researchers caution that the association between in-person instruction and a reduced risk of contracting COVID-19 during the study duration is weak (22% relative difference; 95% CI -77%-33%). There was a positive correlation between in-person instruction and quality of life (3%; 95% CI 2%-4%; and teacher satisfaction (10%; 95% CI 8%-11%); There was much doubt about whether or not COVID-19 infection was linked to the style of instruction. The well-being of college students appears to suffer due to the shift from face-to-face to online instruction. They did not find clear evidence of an association between COVID-19 infection and teaching modality for students in higher education. Still, their findings indicate that shifting from in-person to online teaching may negatively impact students’ well-being. Consequently, we saw the mental health condition of university graduates deteriorate during the pandemic.

There is a high chance that the mental health conditions of the students may further exacerbate after the COVID-19 period because of the worsening teacher-student relationship and burgeoning academic distress. Thus, it is important to critically assess the teacher-student relationship in the context of COVID-19, especially in developing countries such as Bangladesh. Furthermore, teachers can also play a significant role in repairing the damage done to higher education in developing countries. Unfortunately, little research has been conducted on the teacher-student relationship at the tertiary level. To date, no study has been found on the impact of COVID-19 on teacher-student relationships at the university level. Therefore, this study will significantly contribute to the knowledge world regarding the paradigm of COVID-19 in higher education in developing countries.

## Methodology

### Overview of proposed methodology

**Fig 1:**
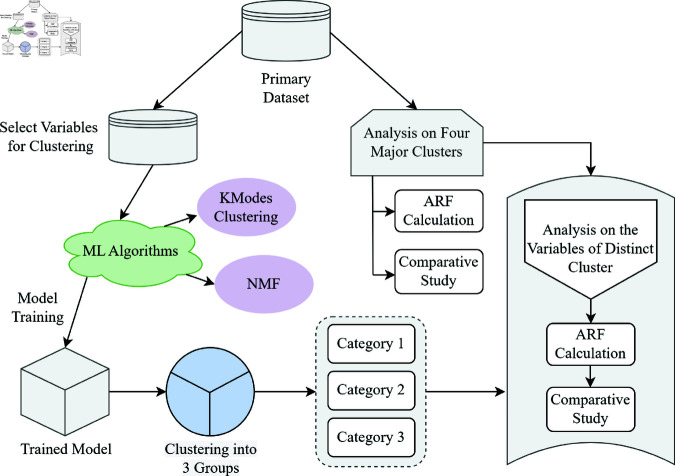
Overview of proposed methodology from data collection to analysis. This methodology finds the change in the teacher-student relationship due to the COVID-19 pandemic.

To find the relationship between teacher and student and how COVID-19 as an event influenced the dramatic change, we use AI-based tools with Explanatory Data Analysis (EDA) [20]. A step-by-step top-down approach to our proposed methodology is present in Fig 1. Primary raw data was collected by a questionnaire that captures the answer of a single variable three consecutive times one for before COVID-19, one for during COVID-19 and another one for after COVID-19. There is a major reason behind this data collection. Because we could only see the disruption in teacher-student interaction during the pandemic and sense its negative impact on the teacher-student relationship after that certain period of time. Thus, we could not conduct the survey before or during COVID-19 and had to rely on retrospective data collection. We collected data three months after classes resumed following the pandemic. As, data collection time was within few months following the pandemic, we suppose respondents provided reasonably accurate information. After collecting the primary data, we cleaned the dataset and finalized it encoding the questions to unique code for each major group. The final dataset undergoes a basic preprocessing technique to be standardized for analysis by AI-based tools. The whole analysis is mainly divided into two major parts. Firstly, we perform a comparative study among the variables of the major groups by proposing a new technique called Absolute Rate of Fluctuation (ARF). In addition to the major group change analysis, the variables inside individual major groups are also considered in the analysis. Secondly, we use the unsupervised machine learning algorithm K-Modes clustering [21] and Non-negative Matrix Factorization (NMF) [22] to cluster the data into several groups considering the internal correlation of the variables suggested by the Elbow method (For K-Modes Clustering). Then we compare this average score achieved by individual variables based on gender, department, and change of time (before covid, during covid, and after covid). Based on the analysis, we make different decisions and find the dimension of the relationship in the three-time domain. The details of the algorithms and proposed ARF method is in Sect.

**Table 1:** Teacher-student relationship questionnaire variables with descriptions.

Question	Pre-Covid	During Covid	After Covid
Taking Class regularly	PCC1	DCC1	ACC1
Conducting their class as per schedule	PCC2	DCC2	ACC2
Opportunity for students to participate spontaneously in class	PCC3	DCC3	ACC3
Adequate preparation before the start of class	PCC4	DCC4	ACC4
Provide adequate reading materials (books / sheets)	PCC5	DCC5	ACC5
Classroom comprehension skills	PCC6	DCC6	ACC6
Role of University Authority to eradicate session Jot	PCC7	DCC7	ACC7
Role of your department teachers to eradicate session Jot	PCC8	DCC8	ACC8
Taking midterm, quiz etc.	PTEA1	DTEA1	ATEA1
How are your teachers evaluating students?	PTEA2	DTEA2	ATEA2
Give feedback in class about your test results	PTEA3	DTEA3	ATEA3
Opportunity to get advice from teachers	PTEA4	DTEA4	ATEA4
How student friendly are your teachers?	PIS1	DIS1	AIS1
What is the status of regular contact with teachers?	PIS2	DIS2	AIS2
How to help your teachers in skill development and career	PIS3	DIS3	AIS3
How much help you get in academic matters outside of class?	PIS4	DIS4	AIS4
What is the role of teachers in your crisis?	PIS5	DIS5	AIS5
How to rely on teachers in stressful or stressful situations?	PIS6	DIS6	AIS6
How satisfied you are with the academic performance of the teachers	PARA1	DARA1	AARA1
How satisfied you are with the research work of the teachers	PARA2	DARA2	AARA2
What an opportunity for students to be involved in teachers’ research work	PARA3	DARA3	AARA3

### Details of dataset

This study considers Bangladesh’s public and private universities as the field of data collection using a questionnaire. Total of 401 participants from both STEM and non-STEM departments where both male and female students provide their information. We have collected data from different universities from 25 December 2022 to 2 February 2023. We took the writing consent from the participants during the data collection process. The details of the variables with their acronyms are in Table 1.

In our dataset, we have collected some demographic information about the students. The demographic information included with ‘Age’, ‘Gender’, ‘University’, ‘Department’. Statistics shows that the mean age of the participants are 22.73 (23), as we mainly take the data from university students. Among the participants 40.25% are male and remaining 59.75% are female. We main conduct survey in four reputed university in Bangladesh, where 50.75% of data are from Hajee Mohammad Danesh Science and Technology University, 24.5% data from Rajshahi University, 22.25% data from Begum Rokeya University, Rangpur, and 2.5% data from Rajshahi University of Engineering and Technology. In the dataset, we found 14 different department combining STEM, and non-STEM departments. We found almost 22% data from sociology, 13.25% from social work. This two are huge non-STEM categories data. Additionally, the dataset contain Psychology 4%, Mechanical Engineering 4.75%, Mathematics 7.5%, Marketing 6.75%, Agriculture 0.75%, Accounting 9.5%, Civil Engineering 1.25%, Computer Science and Engineering 1.25%, Electronics & Communication Engineering 11.75%, Development Studies 6.25%, English 10%, and Economics 1%. In totla, we have found 28% data are from STEM and 72% from non-STEM.

### Dataset participants and survey area

In our dataset, we have collected data from the student parts. We did not interview teachers mainly for three reasons. Firstly, interviewing teachers could potentially lead social desirability bias. Most of the Universities in Bangladesh remained shut and almost all of these did not conduct classes (oﬄine or online) throughout the COVID-19 period. In absence of effective institutional support for students during pandemic, it was expected that teachers would maintain a close communication with their students and support them both academically and emotionally. Therefore, asking teacher whether they maintained regular communication and provided supports to their students could lead to social desirability bias in survey response. Secondly, asking teacher about teacher-student relationship could generate an incomplete or confusing picture of true phenomenon. Student-teacher ratio in Bangladesh is around 23 (considering both public and private university). Even if a teacher maintained communication with only one or two students and truthfully reports that they stayed in touch with students during COVID-19, it becomes challenging to determine what proportion of students had regular communication and received consistent support from their teacher. Thirdly, we did not include teacher interviews because our focus was on students’ experiences and satisfaction, which are central to the teacher-student relationship and cannot be adequately captured through interviews with teachers. Interviewing teacher merely could provide us information regarding the frequency of interaction between teacher and student. However, it is difficult to gauge experience or satisfaction of students during that time. On the contrary, interviewing students could sufficiently provide us frequency and quality of the teacher-student relationship.

### Description of the algorithms used in this study

#### Absolute rate of fluctuation (ARF)

Absolute Rate of Fluctuation (ARF) measures the fluctuation of two individual variables over a different period. In this study, we divided the questionnaire three times based on COVID-19. Every variable under a section is taken for pre-COVID-19, during COVID-19, and after COVID-19. ARF indicates the change of the variables according to the change of time. We need to do some pre-calculation to find the ARFs among the three-time period under a section. Firstly, the following equation calculates the average score obtained by a participant from the variables in a period under a section. It is denoted by the average score of a participant (ASP) in a time under a section.


$$\begin{array}{cccc} & ASP=\frac{\overset{n}{\underset{i=1}{\sum }}X_{i}}{n} & & \\ & where, & & \\ & n=number\;ofvariables\;under\;a\;section & & \\ & X=variable\;of\;index\;i & \end{array}$$ (1)

Then, the average of the ASP is calculated for all the participants in a period of time under a section. The average value of a time period under a section is denoted by the average period score (APS). The equation to calculate APS is below:


$$\begin{array}{c} APS=\frac{\overset{m}{\underset{i=1}{\sum }}{\left(\frac{\overset{n}{\underset{i=1}{\sum }}X_{i}}{n}\right)}_{j}}{m} \end{array}$$ (2)

We can reform the equation from , we can represent APS as,


$$\begin{array}{cccc} & APS=\frac{{\overset{m}{\underset{j=1}{\sum }}(ASP)}_{j}}{m} & & \\ & where, & & \\ & n=number\;of\;variables\;under\;a\;section & & \\ & m=number\;of\;total\;observations & & \\ & X=variable\;of\;index\;j & \end{array}$$ (3)

We calculate the APS for each period under every section for further analysis. We need the degree of measurement to find the ARF between two different periods. For five scale Likert Chart, the degree is five; for the three scales, the degree is 3. Finally, ARF is come out by .


$$\begin{array}{cccc} & ARF=\frac{\left|(APS_{1})-(APS_{2})\right|}{c}*100 & & \\ & where, & & \\ & APS_{1}=average\;of\;period\;1 & & \\ & APS_{1}=average\;of\;period\;2 & & \\ & C=degree\;of\;measurement & \end{array}$$ (4)

### K-Mode clustering

K-Modes clustering is an unsupervised ML algorithm that divides an unlabeled dataset into distinct clusters to create the level of the data. The cluster is determined by the number K, where K is the number of pre-defined clusters that must be created [23]. The k-modes algorithm replaces cluster means with the modes of different features accordingly. It uses a frequency-based method to update modes during the clustering process to deal with category objects and reduce the clustering gradient descent. K-modes cluster uses a randomly selected starting cluster center (modes) as a seed. This leads to problematic clustering results that often depend on the choice of the beginning cluster center and the possibility of getting cluster structures that don’t repeat individuals [24]. The k-mode technique extends the K-mean pattern to cluster categorical data by eliminating the limitation forced by K-means [14] following modification:

 ∙  Using simple match dissimilar evaluate or hamming the distance used for the categorical data object.

 ∙  Change means of cluster modes


$$\begin{array}{c} d(x,y)=\overset{f}{\underset{i=1}{\sum }}\delta (x_{j},Y_{j}) \end{array}$$ (5)

d (x, y) gives equal significance to every kind of attribute. Let Z be a set of categorical data objects described by categorical attributes, A1, A2 . . . . . .. Am. while the above is used because the dissimilarity determine for categorical data objects, the cost function become


$$\begin{array}{c} C(Q)={\left(\underset{i}{\sum }=1\right)}^{n}d(Z_{i},Q_{i}) \end{array}$$ (6)

Where *Z_i_* is the ith element and *Q_i_* is the near cluster center of *Z_i_*. The K-modes technique minimizes the cost Function defined in .

**Fig 2:**
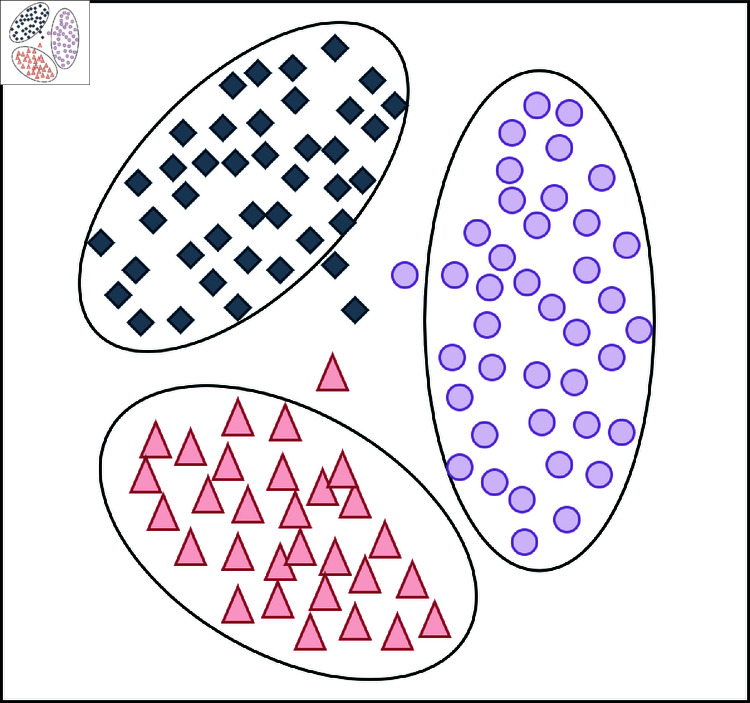
Clustering view of K Modes clustering.

The following processes make up the K-modes, which presuppose that the information regarding the number of probable groups of data (i.e., K) is accessible:

- Step 1. Choose data objects randomly to create K clusters; for each cluster, choose the center of the first cluster.
- Step 2. Assign data objects to the cluster whose cluster center is near it according to the cost function.
- Step 3. Update each cluster’s K cluster based on the distribution of data objects and calculate the K clusters’ most recent modes.
- Step 4. Repeat steps 2 to 3, awaiting no data object has changed cluster relationship.

**Elbow Method:** In cluster analysis, the elbow method is a heuristic for estimating the total number of clusters in a dataset [25]. To determine the optimal number of clusters, we plot the explained variation against k and take the elbow of the curve. The elbow approach determines the optimal number of clusters in k-means clustering. The elbow approach plots the value of the cost function by changing the value of k. Parameters in other data-driven models, like the number of primary components representing a data set, can be selected in the same way. Instances get closer to their cluster centers, the number of component examples in each cluster reduces, and the average distortion decreases as k increases [26]. If we continue to cluster the data past the elbow, the value of k at which the improvement in distortion drops down the highest, we will likely miss important patterns.

**Fig 3:**
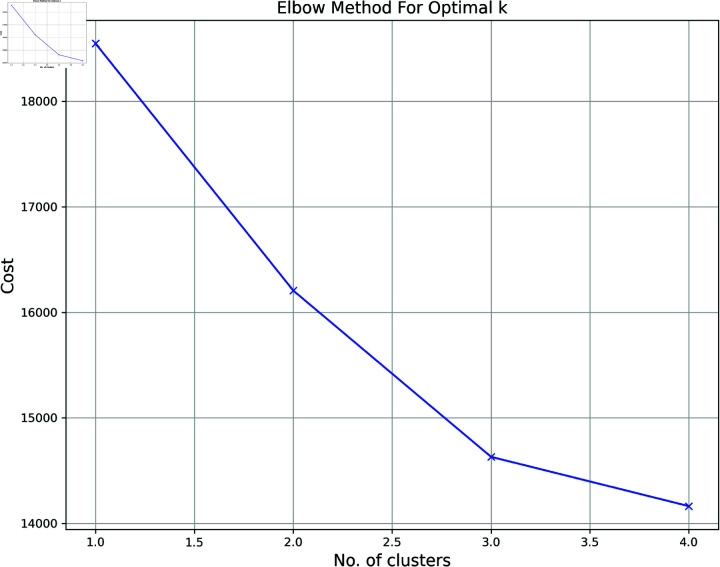
Optimal K value identification recommended by Elbow method.

### NMF

NMF is a relatively new way of reducing the dimensionality of our data into a linear combination of bases [27]. Non-Negative Matrix Factorization is a cutting-edge feature extraction algorithm. When there are many attributes and the attributes are unclear or have poor predictability there, NMF is helpful. It can create significant patterns, subjects, or themes by combining attributes. NMF has a non-negative constraint; it can be used to represent data with non-negative features quite well [28]. It constructs sparse bases and weightings that assume underlying structure to the data and is used as a dimensionality reduction pre-processing step in classification, regression, clustering, and other data mining strategies. NMF enhances compression and interpretability due to its parts-based and sparse representation from the nonnegativity or purely additive constraint [29]. One obvious disadvantage of using this for data representation is that it misses the geometric structure of the data.

## Result and discussion

**Fig 4:**
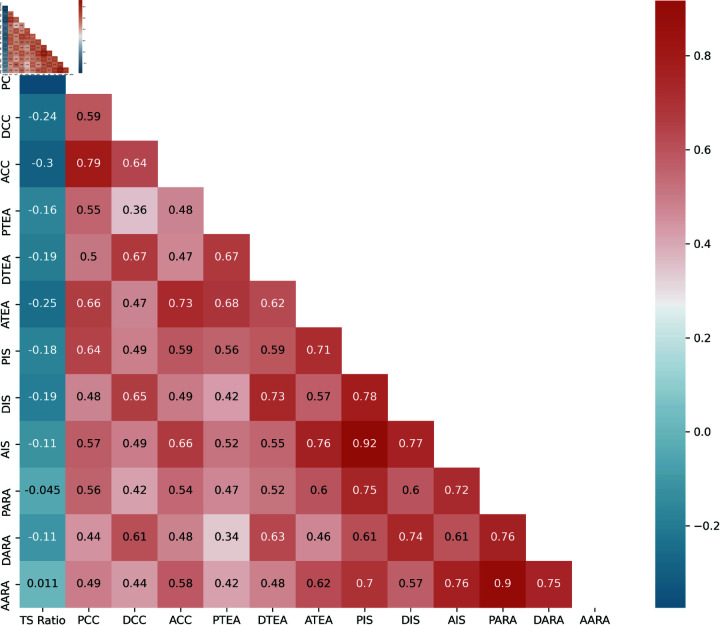
Heatmap of the variables to represent the correlation among the variables in three time period.

To determine the relationship between teacher and student, we divide the variables into four different major groups (Class Conducting (CC), Taking Exam and Assessment (TEA), Interaction and Support (IS), and Academic and Research Activity (ARA)). The variables of each group are interrelated with each other’s also the variables of different groups are correlated. We use Pearson correlation, and the correlation values represent by a heatmap in Fig 4. Different intensity of the color represents the strength of correlation. If we look at the heatmap from a different point of view, we will get some interesting scenarios hidden inside the map. The variables of pre-COVID-19 have a good correlation among themselves. Also, the correlation during and after COVID-19 variables are correlated in a good manner among themself. But the relationship between pr-COVID-19 and COVID-19 is not as good as before. It indicates the fluctuations of the relationship during this pandemic. After and pre-COVID-19 variables have a good correlation that indicates the fallen situation is being recovered gradually. The proposed methodology analyzes and represents the stories in the next sections.

The teacher-student ratio (TSR) always shows negative correlation with the pre, during and post Covid variables. When there is a huge different between the number of teachers and students (High TSR), the class conducting situation, assessment and exam, personal interaction, and involvement of research and academic activities effects negatively.

**Table 2:** Achieving score and ARF for four different clusters of the variables in pre-COVID-19, during COVID-19 and after COVID-19 period.

Major Groups	Pre Covid	During Covid	After COVID-19	Pre to During COVID-19 (%)	During to After COVID-19 (%)	Pre to After COVID-19 (%)
CC	3.59	2.63	3.58	19.16	18.93	0.23
TEA	3.44	2.05	3.40	27.84	27.15	0.69
IS	3.18	2.47	3.23	14.21	15.30	1.09*
ARA	3.10	2.41	3.15	13.92	14.65	0.74*

### Analysis to find the impact of COVID-19 on teacher student relationship

**Fig 5:**
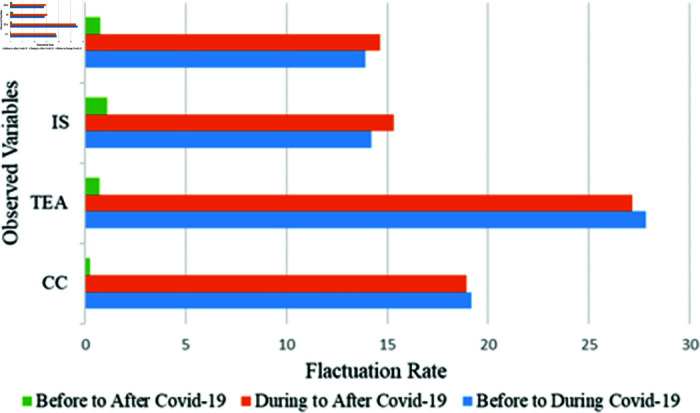
The change of the four major variables in the three-time period represents the fluctuation of the relationship between teacher-student.

To find the relationship between the teacher-student and the fluctuations that appeared due to COVID-19, Table 2 represents the result of four major groups. All the variables are seen to be down from the pre-COVID-19 situation to during COVID-19 situation. But after COVID-19, all of them recovered in a significant manner. Table 2 appears that the fluctuation rate of Taking Exam and Assessment (TEA) is 27.84% in pre to during covid, which is maximum than others. During COVID-19 situation, it also recovered to 27.15%. But there remains 0.69% lacking. But in the Pre-COVID-19 situation, we can see that Academic and Research Activity (ARA) and Interaction and Support (IS) get minimum values of 3.10 and 3.18, respectively. It indicates that before COVID-19, the personal interaction and involvement of the students in their teacher’s research activities are not in a good stage. But the situation has changed and improved after the COVID-19 situation. The Class Conducting (CC) and TEA have an average score of 3.59 and 3.44, respectively, which is better than the other major groups. Though there was a simple lack, they have recovered after the COVID-19 situation. But the fluctuation in COVID-19 is really going down in a significant manner. Due to COVID-19, a great gap was created, and both teachers and students are concerned about the issue. Though TSR is a major issue in academic, everyone is conscious and take the necessary step willingly to recover the gap. It indicates that the COVID-19 situation has a negative impact on the teacher-student relationship and creates a positive impact in some sectors than previously. Fig 5 represents the fluctuations by a bar diagram.

Now, focus on the individual variables of each cluster. The eight CC variables show a dramatic picture in the three periods in Table 3. Before the COVID-19 period, most of the variables have a good score except CC7 and CC8, which represent the responsibility of the teachers and university authority to mitigate the session jot. In Bangladesh, session jot is a common problem in most public universities, and private university students are unknown on this issue. Also, the authority is irresponsible in this segment. Also, the teachers ignore this problem before COVID-19. Due to a lack of interaction, and online classes and exams, these two variables’ scores fall significantly. But, the improvement of these two variables after COVID-19 is remarkable and dramatic. The teachers and university authorities rank it as one of the major problems, and they take some necessary steps to mitigate it. The score rises 5% in CC7 and 3.87% in CC8. In CC5, the capability of the teachers to understand the students in class is stable at three separate times. It indicates that online classes can be an alternative when such kind of events as COVID-19 arise up. But, the variable CC1 means the regular class conducted by the teachers affect more negatively than others. From pre-COVID-19 to during COVID-19, is fall at 31.23% rate, and after COVID-19, it recovers 28.72% but still 2.51% lacking in this part. It is alarming that the rate of regular students in class is decreasing after the COVID-19 period. Students are losing their interest in class after the situation. Also, the materials supply of the class decreased after COVID-19, and teachers were not taking good preparation before class both before COVID-19 and after COVID-19, as scored by the participants of this study. Overall analysis shows that COVID-19 has a mixed effect on the variables of this CC segment.

**Table 3:** Achieving score and ARF for the variables of CC clusters in pre-COVID-19, during COVID-19 and after COVID-19 period.

Conducting Class (CC)	Pre Covid	During Covid	After COVID-19	Pre to During COVID-19	During to After COVID-19	Pre to After COVID-19
CC1	3.93	2.37	3.80	31.23	28.72	2.51
CC2	3.48	2.47	3.41	20.28	18.85	1.43
CC3	4.12	2.91	4.00	24.30	21.85	2.45
CC4	3.66	2.76	3.51	16.16	15.09	1.07
CC5	3.81	3.03	3.66	15.65	12.63	3.02
CC6	3.85	2.91	3.81	18.65	17.94	0.71*
CC7	2.80	2.15	3.05	12.89	17.89	5.00*
CC8	3.19	2.46	3.39	14.63	18.50	3.87*

**Fig 6:**
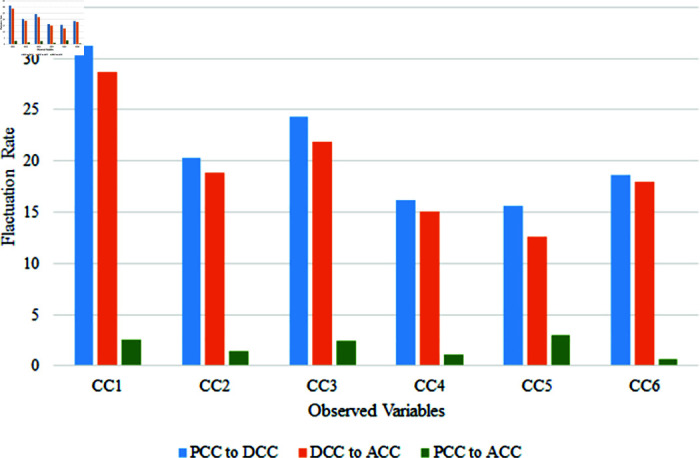
The fluctuations of class conducting variables in pre COVID-19, during COVID-19 and after COVID-19 period.

**Fig 7:**
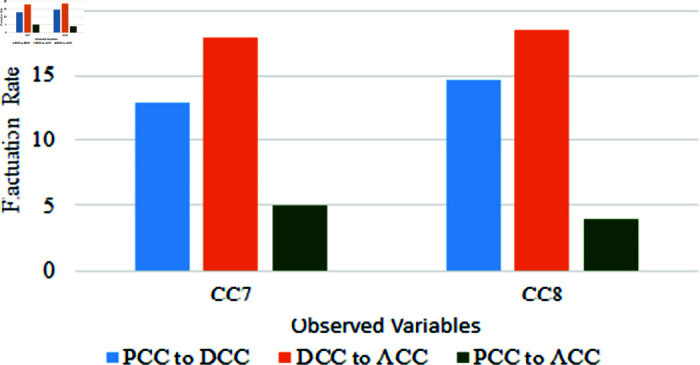
Relationship of the variables of CC in pre COVID-19, during COVID-19 and after COVID-19 period.

The fluctuation rate before COVID-19 to COVID-19, during to after COVID-19, and before COVID-19 to after a bar chart visualizes COVID-19 in Fig 6, which shows the fluctuation rate of three segments of the variables CC1 to CC6. All of these six variables’ recovery rate is still below the falling rate. Another two CC7 and CC8 variables of this segment represent separately in another figure in Fig 7. These two variables rise up then another variable, and this figure represents the positive impact of COVID-19.

**Fig 8:**
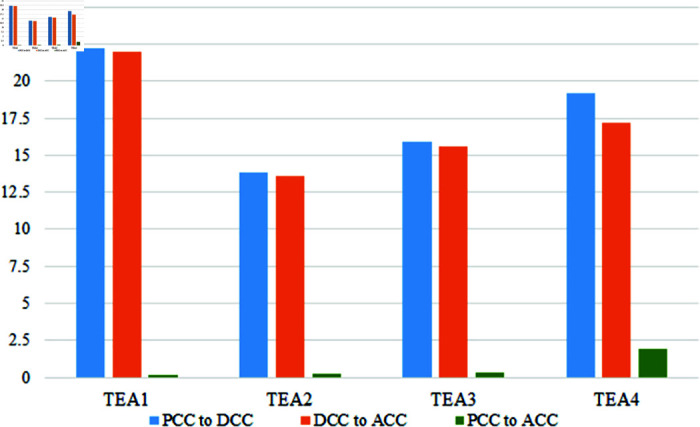
Changes of the variables of TEA in three time periods.

Variables of the TEA cluster achieve low scores than CC clusters variables in all of the three-time domains. Table 4 indicates the achieving score of the variables in pre-Covid and after-COVID-19 are almost the same. That means the relationship in this segment is stable now but still has some gap. Due to online-based exams and assessments, the variables score in during COVID-19 are not at a good level. Timely class tests and midterms fluctuate more downward than others. After COVID-19, it is in his previous state through the achieving score in any state is not satisfactory. Teacher feedback on assignments, midterms, and quizzes is really at a low level now and then. Students can not get the obtained marks timely, which negatively affects the final exam, and it is a major factor in getting a bad academic result. TEA4 scored better than other variables, indicating that students had a good scope to get suggestions from teachers. It fluctuated badly during COVID-19 at 19.15% rate and recovered at 17.19% rate; still, the gap between the two states is 1.96%. Fig 8 shows the fluctuation of the three individual times.

**Table 4:** Achieving score and ARF for the individual variables of TEA clusters in pre-COVID-19, during COVID-19 and after COVID-19 period.

Taking Exam and Assessment (TEA)	Pre Covid	During Covid	After COVID-19	Pre to During COVID-19	During to After COVID-19	Pre to After COVID-19
TEA1	3.43	2.32	3.42	22.21	22.01	0.20
TEA2	3.57	2.87	3.55	13.86	13.58	0.28
TEA3	2.98	2.19	2.97	15.89	15.59	0.30
TEA4	3.78	2.82	3.68	19.15	17.19	1.96

**Fig 9:**
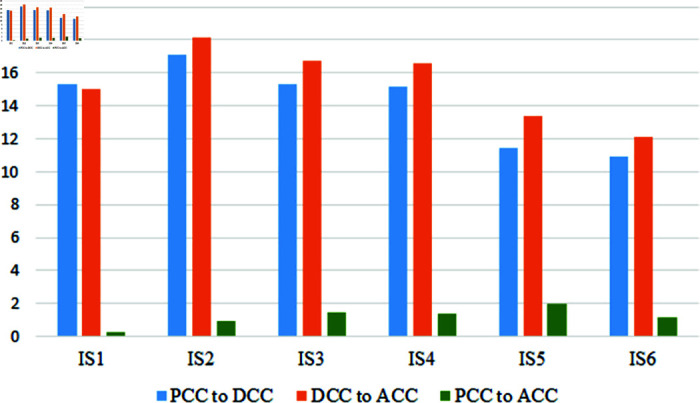
Fluctuation of the IS variables in three time periods.

Interaction and Support (IS) is one of the major clusters in teacher-student relationships. The previous two cluster (CC, TEA) is directly involved with the teachers, who take their course directly. But this cluster is not based on the context. Any teacher at the university can be considered as the analysis factor for these variables. The score achieved by individual variables is average in Table 5, but IS4 means the help of the teachers out of academics gets the minimum score of 2.89. The maximum score achieved in this cluster by the variable IS1 is 3.61. It indicates the teachers are student-friendly and its score after COVID-19 is 3.60 which indicates the stability of this relationship. Communication with teachers fell during the COVID-19 period as the lockdown and the university was shut down for a long time. Though the strength of the relationship between teachers and students is not stronger in this cluster almost all the variable score gets up. This indicates the positive impact of COVID-19 on teacher students’ relationships. In this critical period like COVID-19, teachers are more concerned about their students. They support them mentally as well as sometimes economically. The growth of IS5 variables is more than other variables that can define this claim. Also, students are getting more support to develop skills and getting proper guidelines from teachers. Support of the teachers outside the classroom also increases at a 1.40% rate. This improvement is amazing for building a good relationship in these types of sectors, where the strength of the relationship improves the quality of education. The rate of fluctuation is represented in Fig 9.

**Table 5:** Achieving score and ARF for the individual variables of IS clusters in pre-COVID-19, during COVID-19 and after COVID-19 period.

Interaction and Support (IS)	Pre Covid	During Covid	After COVID-19	Pre to During COVID-19	During to After COVID-19	Pre to After COVID-19
IS1	3.61	2.85	3.60	15.29	14.99	0.30
IS2	3.25	2.39	3.29	17.14	18.11	0.97*
IS3	3.19	2.42	3.26	15.29	16.74	1.45*
IS4	2.89	2.13	2.96	15.19	16.59	1.40*
IS5	3.05	2.47	3.14	11.43	13.38	1.95*
IS6	3.09	2.54	3.15	10.93	12.08	1.15*

**Fig 10:**
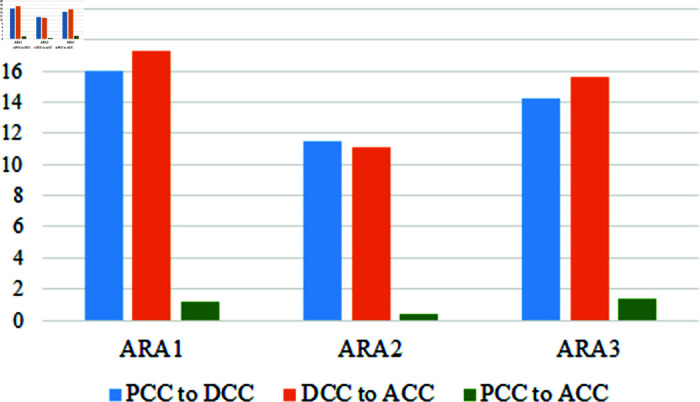
Relatonship of the ARA variables in three different time period.

The last cluster is Academic and Research Activities (ARA). It contains three variables, and the achieving score is not satisfactory in pre, during, and after the COVID-19 period in Table 6. Though students have some idea and understanding of the academic activities of their teachers, there is a large gap in the research domain. Students are slightly satisfied with their teacher’s research work but think the scope of involvement in it is unavailable. During the COVID-19 period, all three variables fall. In this segment, we can see the improvement in the score of the variables. It indicates that students are getting interested in research work. A graphical representation of this segment is in Fig 10.

**Table 6:** Achieving score and ARA for the individual variables of IS clusters in pre-COVID-19, during COVID-19, and after the COVID-19 period.

Academic and Research Activity (ARA)	Pre Covid	During Covid	After COVID-19	Pre to During COVID-19	During to After COVID-19	Pre to After COVID-19
ARA1	3.38	2.58	3.44	16.04	17.24	1.20*
ARA2	3.10	2.52	3.08	11.48	11.12	0.35
ARA3	2.85	2.14	2.91	14.24	15.59	1.35*

### Gender and academic subject wise distribution

To show the fluctuation of the relationship we use K Mode Clustering and NMF methods that divide the students into three distinct cluster namely Less Fluctuation, Middle Fluctuation and High Fluctuation of relationship. Gender is considered as a major factor to determine the fluctuation in the three cluster. In Less Fluctuation, male is 34.86% which represents 15.89% of the total male student. In Medium Fluctuation, male is 64.58% which capture almost 38.91% of total male student. Also, in High Fluctuation group, 73.47% are male which 45.18% of the total male students is. On the other hand, in Less fluctuation, female is 65.14% which is 44.09% of the total female. At the same time, in Medium Fluctuation, female is 35.42% which represents the 31.42% considering the total female. In High fluctuation of changes, 26.53% are female which represents 24.22% of the total female students.

The above Table 7 indicate, in less fluctuation, the percentage of female is higher than the male students. It indicates that due to COVID-19, teacher-student relationship changes in Less Fluctuation group like a way that the percentage of female is higher than male. But in medium and high fluctuation group, we get an opposite scenario. Percentage of female is lower than male in these two groups. It shows that, due to COVID-19 the relationship fluctuation of female is better than male fluctuation rate.

**Fig 11:**
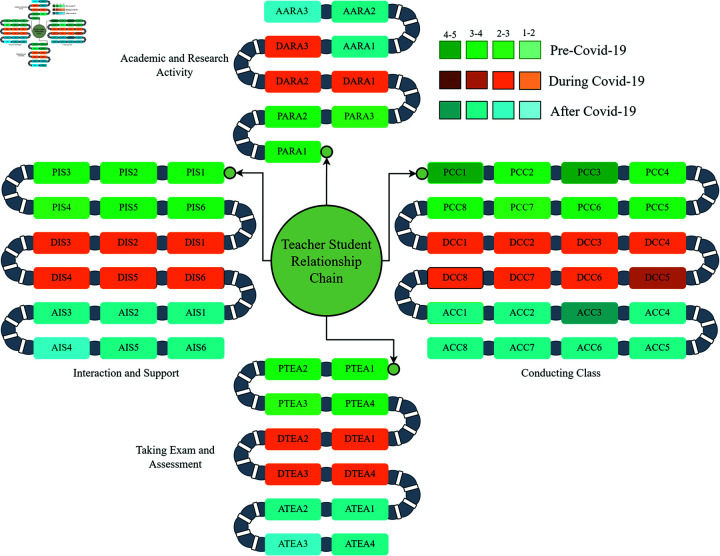
Chain representation of teacher-student relationship.

If the analysis undertakes based on four major groups, we get an interesting story. We divide the above table into different parts based on fluctuation that shows in Less Fluctuation for gender variation. Table 8 displays the average and individual values of the major clusters, which are CC, TEA, IS, and ARA, that have a significant change in gender perspective from Pre Covid to During, During to Post COVID-19 and also pre-post situation. For the major four clusters, the average value for male is 3.83 and for female is 4.00. But During COVID-19, it is fluctuated for both male and female and represents 2.83 and 3.13 accordingly. In Post COVID-19 situation, the average score for male is 3.89 and for female is 4.11. It indicates the teacher-students relationship for post COVID-19 is higher than pre and during COVID-19 situation.

**Table 7:** Distribution of Male and Female in a different group of fluctuation.

	Less Fluctuation	Medium Fluctuation	High Fluctuation
% Of total observation	27.25	36.00	36.75
Male	34.86(15.89)	64.58(38.91)	73.47(45.18)
Female	64.14(44.09)	35.42(31.67)	26.53(24.22)

If we consider this in ARF rate, we can see the straightness of relationship for male during COVID-19 decreased by 19.4% on average. After COVID-19, the relationship rebuilt and it is 20.60%. The strength of the relationship increased from before to after COVID-19, with a percentage of 1.2. For female, the relationship of before COVID-19 was fluctuated by 17.4 percent and after COVID-19, it is recovered by 19.6 percent which represents the increasing strength of relationship with a percentage of 2.2%.

When we consider individual clusters in class Conducting (CC) for both male and female, the strength of the relationship increases to 1.00 and 1.80, respectively. The increasing rate of female is little more than male and it is .08%.

In TEA cluster, the percentage between male and female increased gradually but the percentage of female increased significantly than male. The increasing percentage between male and female is 2.4. In the IS cluster, however, the percentage increases for both male and female is nearly equal. On the other hand, in the ARA cluster, however, the percentage increased for both male and female, but the Closeness in Academic and Research Activity increased more for male than female.

Dividing the respondents into three clusters, if we consider the achieving score and average rate of fluctuation from the above table, we can consider that the main cluster in a broad sense, such as CC, TEA, IS, and ARA, it focuses on the achieving average score of males being much lower than female both before, during, and after COVID-19. When the average rate of fluctuation in Pre-COVID-19 and During COVID-19 was examined, it was noticed that the fluctuation experienced by males was not the same as that experienced by females.

For example, in Class Conducting (CC), the fluctuation for males is 20.06%, and for females, it is only 16.4%. Meanwhile, During COVID-19 to Post COVID-19 fluctuation situation for male, the it increases from 20.06 to 21.6% and represents 1.0% of increase. On the other hand, situation for female represents that the fluctuation in Pre to during COVID-19 which was 16.4% increases to 18.2% in post COVID-19 situation and represents 1.8% of up warding situation than during COVID-19. Overall, it represents the situation in fluctuation of female is better than male. The relationship of female with teacher was better in before COVID-19 and after COVID-19 the situation increase.

**Table 8:** Gender wise achieving score for less relation fluctuation group.

Major Groups	Pre Covid		During Covid		Post Covid		Male			Female		
	M	F	M	F	M	F	P_1_-D	D-P_2_	P_1_-P2	P_1_-D	D-P_2_	D-P_2_
CC	4.01	4.22	2.98	3.40	4.06	4.31	20.60	21.60	1.00	16.40	18.20	1.80
TEA	3.81	4.08	2.37	2.62	3.83	4.22	31.80	29.20	0.40	29.20	32.00	2.80
IS	3.75	3.89	3.05	3.25	3.85	4.00	14.00	16.00	2.00	12.80	15.00	2.20
ARA	3.75	3.82	3.03	3.23	3.83	3.89	14.40	16.00	1.60	11.80	13.20	1.40
Avg	3.83	4.00	2.86	3.13	3.89	4.11	19.40	20.60	1.20	17.40	19.60	2.20

**Table 9:** Gender wise achieving score for medium relation.

Major Groups	Pre Covid		During Covid		Post Covid		Male			Female		
	M	F	M	F	M	F	P_1_-D	D-P_2_	P_1_-P2	P_1_-D	D-P_2_	D-P_2_
CC	3.42	3.56	2.58	2.61	3.59	3.50	16.80	20.20	3.40	19.00	17.80	1.20*
TEA	3.59	3.57	1.92	2.11	3.31	3.45	33.40	27.80	5.60*	29.20	26.80	2.40*
IS	2.88	3.31	2.17	2.51	3.14	3.35	14.20	19.40	5.20	16.00	16.80	0.80
ARA	2.97	3.22	2.10	2.40	3.32	3.25	17.40	24.40	7.00	16.40	17.00	0.60
Avg	3.22	3.42	2.20	2.41	3.34	3.39	20.40	22.80	2.40	20.20	19.60	0.60*

Similarly, this Table 9 shows gender-specific achieving scores for the Medium Relationship Fluctuation group, in which, due to COVID-19, students’ teacher-student relationships fluctuated similarly to the Less Fluctuation group. The average values for males and females in the four major clusters are 3.22 and 3.42, respectively. Throughout COVID-19, the average value fluctuated between 2.20 and 2.41 for males and females. In terms of percentage, males recover from relationships faster than females. The rebuilt male relationship increased by 2.40% where it is. 60 points for female students. Considering the four main clusters, in CC, the fluctuation rate for male students from before to during and after COVID-19 represents 16.8 and 20.2 percent, respectively, an increase of 3.40 percent from before to after COVID-19. The pre-COVID-19 fluctuation for female students is 19%, but the post-COVID-19 situation is drastically different than the pre-COVID-19 and it 17.8%. This demonstrates that compared to male females in the Medium fluctuated group, the rebuilding relationship is far superior to females.

However, TEA represents that male and female relationship are consistently better in pre-COVID-19 times but fluctuate in post-COVID-19 times. In IS, the fluctuation of the relationship of the pre-COVID-19 condition for males is much better than the post-COVID-19 period, but the changes for females are always the same as the pre-post condition. However, in ARA, there have been significant improvements in male relationships, with a constant 7% improvement from pre to post. Notwithstanding, despite an improvement of.60%, it remains unchanged for women.

The High Relationship Fluctuation group’s gender-specific achievement scores are shown in this Table 10, where the average male and female scores for the four major clusters are equal to those obtained during the COVID-19 with the pre-post scenario. The restored scenario for males is conspicuously different from that for females, where the latter are down 1.6% from pre-COVID-19. The average fluctuation values for the teacher-student relationship in each of these four major clusters are lower than they were during the pre-post control period for both male and female students. In CC, the improvement in relationship fluctuation rose for men by 0.80%, while the picture is different for women. That means, after COVID-19, the teacher-student relationship for males improved except for ARA but for females, compared to pre COVID-19 situation, their relationship didn’t improve like males. In the TEA and IS clusters, the rebuilt relation for males increased slightly from the pre-during to the post-COVID-19 period, but the recovery for females did not occur as quickly as for males. In ARA, relationships are constantly improving compared to other clusters, while female relationships remain unchanged.

**Table 10:** Gender wise achieving score for high relation fluctuation group.

Major Groups	Pre Covid		During Covid		Post Covid		Male			Female		
	M	F	M	F	M	F	P_1_-D	D-P_2_	P_1_-P2	P_1_-D	D-P_2_	D-*P_2_*
CC	3.27	3.03	2.04	2.11	3.31	2.89	24.60	25.40	0.80	18.40	15.60	2.80
TEA	3.05	2.74	1.47	1.57	3.10	2.69	31.60	32.60	1.00	23.40	22.40	1.00
IS	2.77	2.35	1.69	1.74	2.82	2.30	21.60	22.60	1.00	12.20	11.20	1.00
ARA	2.53	2.23	1.71	1.67	2.42	2.16	16.40	14.20	2.20*	11.20	9.80	1.40
Avg	2.91	2.59	1.73	1.77	2.91	2.51	23.60	23.60	0.00	16.40	14.80	1.60

**Table 11:** Distribution of STEM and Non-STEM in different groups and their fluctuation.

	Less Fluctuation	Medium Fluctuation	High Fluctuation
STEM	16.51(16.51)	47.71(36.11)	35.78(26.53
Non-STEM	31.27(83.49)	31.61(63.89)	37.11(73.47)

The aforementioned Table 11 describes how relationships vary by gender based on different fluctuation situations, such as Less, Middle, and High. Here, this Table 11 shows the distribution of STEM and Non-STEM in different groups of fluctuation. In STEM, 16.51% of students experienced “Less Fluctuation,” making up 16.51% of all STEM students. Among non-STEM students, 31.27% were in the “Less Fluctuation” group, representing 83.49% of all non-STEM students. In the “Medium Fluctuation” group, 47.71% of STEM students belonged to this category, accounting for 36.11% of all students. Non-STEM students made up 31.61% of all students in this group, with 63.89% of all students classified under “Medium Fluctuation”. In the“High Fluctuation” group, 35.78% of STEM students were included, contributing to 26.53% of all students. Meanwhile, 37.11% of non-STEM students fell into this category, representing 73.47% of all non-STEM students.

For reliability and validity test, we have used test-retest reliability using pearson correlation. In the Fig 4, the heatmap represent the coefficient of the pearson correlation among the variables. It indicates a positive and consistance values in most of the cases. Additionally, we have divided our dataset into STEM and non-STEM and perform ‘Split-Half Reliability’ test. We have found almost 0.90 values of ‘Spearman-Brown Coefficient’ that indicates excellent reliability of our dataset.

### Discussion

This study investigates the relationship between teacher-student and the impact of COVID-19 on this relationship. After analyzing all the groups and the variables of each group, we got mixed opinions from the participants. All the values of the variables go down during COVID-19 time, which indicates the relationship effect of the event COVID-19. But, after the event, everyone tried to rebuild the relationship to make it consistent compared to the pre-COVID-19 situation. Some of the variables go in the stable position, and most of the variables cannot recover till now. In some groups like CC and TEA, most variables fluctuate negatively, and after the COVID-19 situation, it still has some gap compared to the pre-COVID-19 situation. But IS and ARA groups show different findings. The COVID-19 effect these two groups positively. Also, the participants are divided into three major groups using unsupervised machine learning algorithms KModes Clustering and NMF. Then gender and STEM-wise analyses perform on the three individual clusters. The findings show in terms of female students, the fluctuation is minimum than male students. Generally, the relationship between teachers and female students is better than between male students. Female students are much more interested in conducting class and exam assessments. On the other hand, the male students build their relationship with IS and APA groups variables. Both teachers and students are working to fill the gap, yet there are still fluctuations. The study suggests some findings to minimize those gaps and favorable actions from involved parties will be helpful in this regard.

## Conclusion and future work

The primary goal of this research is to examine the impact of COVID-19 on the teacher-student relationship in higher education within a developing country like Bangladesh. We formulated the key concepts, designed a questionnaire, and collected data from students across various universities and academic disciplines. After initial preprocessing, the variables were clustered into four major segments, and we analyzed the relationships across three time periods: before, during, and after COVID-19. We propose a novel methodology, the Absolute Rate of Fluctuations, to detect changes in relationships over specific time periods. This method is effective in identifying fluctuations in relationships for specific factors across different timelines. Additionally, we employed two unsupervised machine learning algorithms to cluster the data into distinct groups based on their internal characteristics. The analysis reveals that COVID-19 significantly impacted teacher-student relationships. During the pandemic, the relationship often deteriorated, with students becoming disconnected from their teachers and experiencing mental stress. Post-pandemic, efforts were made to rebuild these relationships, with many successfully restored or even strengthened compared to pre-pandemic levels. However, some gaps persist, prompting the implementation of various policies aimed at fostering stronger relationships than before.

This study primarily focuses on academic variables influencing teacher-student relationships. The proposed methodology also has the potential to facilitate empirical analysis of other social relationships and their changes during disruptive events.
